# ALT: A Multi-Faceted Phenomenon

**DOI:** 10.3390/genes11020133

**Published:** 2020-01-27

**Authors:** Aurore Sommer, Nicola J. Royle

**Affiliations:** Department of Genetics and Genome Biology, University of Leicester, Leicester LE1 7RH, UK; as1000@le.ac.uk

**Keywords:** alternative lengthening of telomeres, cancer, telomere-variant-repeat, break-induced telomere synthesis, therapy

## Abstract

One of the hallmarks of cancer cells is their indefinite replicative potential, made possible by the activation of a telomere maintenance mechanism (TMM). The majority of cancers reactivate the reverse transcriptase, telomerase, to maintain their telomere length but a minority (10% to 15%) utilize an alternative lengthening of telomeres (ALT) pathway. Here, we review the phenotypes and molecular markers specific to ALT, and investigate the significance of telomere mutations and sequence variation in ALT cell lines. We also look at the recent advancements in understanding the different mechanisms behind ALT telomere elongation and finally, the progress made in identifying potential ALT-targeted therapies, including those already in use for the treatment of both hematological and solid tumors.

## 1. Telomere Maintenance Mechanisms in Cancer

Telomeres are nucleoprotein complexes that cap the ends of linear chromosomes. Telomeric DNA is made up of hexanucleotides repeats, mainly 5′-TTAGGG-3′, and has a double-stranded portion (ranging from 5 to 12 kb in humans) which terminates in a single-strand overhang of the TTAGGG strand, often referred to as the G-overhang. Telomeric DNA is bound by Shelterin, a protein complex comprising of TRF1, TRF2, TIN2, RAP1, TPP1, and POT1 (as reviewed in [1]). Shelterin prevents chromosome ends from eliciting a DNA damage response (DDR) that is normally triggered by a DNA double strand break (DSB) and protects telomeres from DSB repair by various homology directed repair (HDR) or non-homologous end joining pathways (NHEJ). TRF2 plays a predominant role in telomere protection as it facilitates the formation of the t-loop, a structure created by the G-overhang folding back on, invading, and annealing to the double-stranded portion of the telomere, making the DNA end inaccessible. Unprotected telomeres, that are not bound to Shelterin, result in end-to-end chromosome fusions as the uncapped chromosome ends are recognized as DSBs and joined together by classical or alternative NHEJ pathways [2]. The end-replication problem and subsequent end processing that generates the single stranded, G-rich overhang, means that as somatic cells divide, their telomeres shorten by 50 to 150 base pairs per cell division until a DDR is elicited, followed by arrest in G1 phase and entry into senescence (reviewed in [3,4]). However, this checkpoint can be bypassed by the inactivation of the tumor suppressors TP53 or RB1, following which cells will continue to divide until their telomeres become critically short and enter telomere-induced crisis, a state characterized by extensive genomic instability and cell death [4].

One of the hallmarks of cancer cells is their indefinite replicative potential, made possible by the activation of a telomere maintenance mechanism (TMM). The majority of cancers (85–90%) re-activate the reverse transcriptase telomerase to maintain their telomere length [5,6]. Telomerase is normally only active in embryonic stem cells, germ line cells, and a minority of somatic cells such as epidermal cells, somatic stem cells, and progenitor cells. The remaining minority of cancers (10% to 15%) utilize an alternative lengthening of telomeres (ALT) mechanism [7]. It should however be noted that not all cancers appear to utilize a TMM: Cell lines derived from highly aggressive neuroblastomas were found to start off with long telomeres and experience continuous telomere shortening over 200 population doublings [8]. The minority of cancers that utilize ALT is not evenly distributed across all cancer types. ALT is common in sarcomas and tumors of the central and peripheral nervous systems but rare in common cancers such as breast, colon, and lung cancer and absent from lymphoma and thymoma [3,9]. Among cancers that arise in mesenchymal tissues, 47% of osteosarcomas and 35% of soft-tissue sarcomas (STS) have been shown to utilize the ALT mechanism (ALT positive) [10], although a significant difference between and within subsets of STS types does exist [11]. In liposarcomas (LPS), frequencies of ALT positive tumors range from 0% for well-differentiated LPS (characterized by the amplification of 12q13–15 region) to 80% for pleomorphic LPS, which harbor much more complex genomic alterations [12]. It has been proposed that the prevalence of ALT in STSs could be explained by a tighter repression of telomerase in tissues of mesenchymal origin [13]. The idea that telomerase repression is the reason behind ALT activation has been reinforced by experiments on cells derived from telomerase positive tumors. ALT-like phenotypes have been observed following telomerase inhibition in the colon-derived HCT15 cell line [14], on T-cell lymphoma cells in mice [15] and on human esophageal cancer cells [16]. Moreover, a gene expression signature capable of distinguishing telomerase positive from ALT in cell lines and LPS samples has been uncovered [17]; the signature contains a regulatory signaling network involving repression of the telomerase reverse transcriptase (hTERT in humans).

Of note, some evidence has emerged to suggest ALT may be present in normal somatic cells, first in studies on mice [18] and more recently in primary human cells when a homologous recombination (HR)- based mechanism resembling ALT appeared to be transiently activated as a response to oxidative stress [19] or to X-ray damage [20]. It is also worth mentioning that some malignancies such as neuroblastomas [21] and osteosarcomas [22] exhibit intra-tumoral heterogeneity relative to TMMs, with some cells as telomerase positive and others displaying a typical ALT phenotype.

## 2. Phenotypes Specific to ALT

ALT positive cells possess a high number of extra-chromosomal telomeric repeat (ECTR) DNA, some of which is linear, either single or double-stranded [23]. ECTR can also form circular and double-stranded structures known as t-circles, which are believed to be the end product of telomere trimming, whereby the t-loop is excised following resolution of a recombination intermediate [24,25]. Telomere truncation has also been linked to 5′ C-rich overhangs, which are abundant in ALT cells but found at a low frequency in primary human cells and telomerase positive cells [26,27]. The 5′ C-rich overhangs are proposed to be the result of t-loop excision in the context of excessively long telomeres and are believed to be mediated by the endonuclease XRCC3. A more recent study has suggested that C-overhangs are the result of an unreplicated C-rich strand, following replication fork collapse [28]. The other type of circular ECTR consists of at least partly single-stranded C- or G-circles, if the C- or G-rich strand is intact, respectively. C-circle abundance can be quantified and appears to correlate with the level of ALT activity [29]. It has been proposed that C-circles could act as substrate for telomere elongation in ALT cells, with the telomeric G-overhang annealing to the single-stranded part of the circle and telomere synthesis occurring via rolling-circle replication [30]. The number of C-circles fluctuates during the cell cycle, with an increase observed during S phase before a peak in the late S/G2 [28]. In the same study, the authors proposed a model whereby C-circles are the product of a stalled replication fork, with the lagging strand being excised and circularized to form a C-circle. The presence and persistence of ECTR in ALT cells appears to be linked to a defective cGAS-STING DNA sensing pathway, which was found to be non-functional in some ALT positive cell lines [31].

Some of the ECTR DNA, as well as chromosomal telomeric DNA, is contained within some promyelocytic leukemia (PML) nuclear bodies, which are structures involved in the regulation of various cellular processes [32]. While all PML nuclear bodies comprise of the PML protein and the nuclear antigen SP100, ALT-associated PML bodies (APBs) recruit many of the proteins required for ALT, such as the RAD50/MRE11/NBS1 (MRN) complex [33] and RAD51/RAD52 [34]. APBs are therefore generally thought to play a direct role in telomere recombination. The histone deacetylase HDAC9 is thought to facilitate the nuclear transfer of PML from the cytosol and its depletion results in a decrease in APBs [35]. While C-circles and APBs are deemed to be the most common ALT markers, there are some other phenotypes particular to ALT. Telomere sister-chromatid-exchange (T-SCE) is a post-replication process in which sister telomeres exchange genetic material, although exchange can also happen between homologous and non-homologous telomeres. Unequal T-SCE appears to be frequent in ALT positive cells [36] but extremely rare in normal or telomerase positive cell lines. ALT telomeres are highly heterogeneous in length, but the average length is about double that of most somatic cells where telomeres are maintained by telomerase [37]. ALT telomeres can also display excessively long single-strand G-rich overhangs, with the longest reported measuring 400 nucleotides [38], more than twice the reported overhang length in telomerase positive cells. There is also evidence that ALT positive cells possess highly unusual telomeric structures not found in telomerase positive cell lines. Those comprise single-stranded telomeric DNA in both G- and C-rich regions, unpaired or gapped telomeric DNA, with internal gaps found predominantly in the C-rich strand and t-complexes, highly branched structures with internal portions of single-stranded DNA [39]. Those ALT-specific telomeric DNAs have been hypothesized to be the result of homologous recombination events at telomeres, with the t-complex thought to be an intermediate product similar to a Holliday junction. Those structures have yet to be fully characterized and warrant further investigations, as a better understanding of their biogenesis could further our understanding of ALT mechanisms. Finally, analysis of the hypervariable minisatellite MS32 has shown extreme instability in some ALT positive tumors, [40,41] suggesting there may be increased recombination-driven processes at other locations in the genome of ALT cells.

## 3. Degenerate Variant Repeats: A Role in ALT?

**Figure 1:**
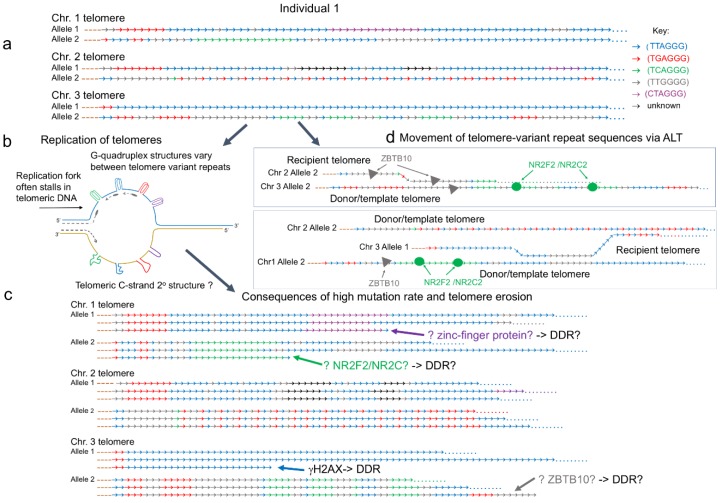
Mutation processes at telomeres. Examples of the interspersion patterns of the canonical (TTAGGG) and telomere-variant repeats (TVRs) commonly found at the proximal (centromeric) ends of human telomeres. (**a**) The diagram represents three telomeres on different chromosomes (Chr. 1 to 3) each with different interspersion patterns in the two alleles. While many telomeres comprise a region of degenerate repeats at the proximal end, some telomeres lack sequence variation in this region, as shown in Chr. 3 allele 1. (**b**) During S phase, the replication fork is prone to pausing or stalling as it passes through telomeric DNA. This is due to secondary structures, such as t-loops (not shown) that must be unwound. In addition, a variety of G-quadruplex structures can form on the G-rich strand. It is not known if the C-strand can also adopt secondary structures that impede replication. These obstacles contribute to high somatic and germline mutation rates that usually result in gains or losses of repeats. (**c**) Telomere lengthening by the break-induced-replication (BIR) mechanism that underlies the alternative lengthening of telomeres (ALT) moves TVRs between telomeres and can result in the distribution of the variant repeats along the full length of the telomeric DNA. The NR2F2 and NR2C2 orphan nuclear receptors can bind to (TCAGGG)_n_ containing telomeres in ALT positive cells. Similarly, ZBTB10 has recently been shown to bind to (TTGGGG)_n_ repeats in ALT positive cells. The binding of these proteins at telomeres may contribute to an altered DNA damage response. (**d**) The replication-driven high mutation rate at telomeres creates mosaicism within normal tissues, cancers, and the germline such that some cells carry mutated telomere interspersion patterns. In addition, the replication-dependent erosion of telomere length results in short telomeres that trigger a DNA damage response (DDR). Telomere erosion may lead to exposure of sequence-variant repeats at the end of telomeric DNA. It is not known whether this triggers specific proteins to bind to the short telomeres, for example NR2F2, NR2C2, ZBTB10, or other zinc-finger containing proteins that may modify the DDR.

The proximal part of human telomeres consists of the canonical telomeric repeat (TTAGGG) interspersed with degenerate or variant repeats [42,43] (Figure 1). The most common group of telomere variant repeats (TVRs) are hexamers with variant nucleotides at positions 1, 2, and 3 of the canonical hexameric repeat, such as TCAGGG, TGAGGG, TTGGGG, and CTAGGG. However, the TVRs usually retain three consecutive G-residues in positions 4 to 6 [42]. There is variation in the type and distribution of TVRs in the proximal portion of telomeres and the interspersion patterns show extensive differences between alleles and between telomeres at different chromosome ends. The high allelic diversity is maintained by rapid turnover of TVRs at the proximal part of telomeres predominantly as a result of slippage, a type of replication error that occurs in regions of tandem repeat arrays [43] and other intra-allelic processes. From family studies, the telomere germline mutation rate has been estimated to be 0.0006 (0.6%) per kb per gamete [44]. Notably, arrays of the (CTAGGG)_n_ are associated with a particularly high male germline mutation rate (20% per gamete) that may be linked to unusual G-quadruplex structures, or secondary structures in the C-rich strand, formed during replication [45]. Telomere repeat arrays also show instability in somatic cells and this increases significantly if the mismatch repair (MMR) pathway is inactive, for example in MMR-defective colorectal cancers [44].

The sequence and interspersion pattern of TVRs in ALT positive cell lines and tumors show even greater complexity [41], arising from the stochastic events that lengthen the telomeres [46]. Telomeres in ALT positive cells undergo complex inter- and intra-allelic mutations not seen in normal or telomerase positive cells, but associated with the strand invasion and copying mechanism that underlies the recombination-based ALT mechanism [47]. The difference in telomeric sequence content between telomerase and ALT positive cells is such that it has been speculated that the ALT status can be determined by whole genome sequencing alone [48].

In WI38-VA13/2RA, an ALT positive cell line transformed by SV40, the variant repeat (TCAGGG) is abundant within telomeric DNA and this recruits the orphan nuclear receptor proteins NR2F2 (also known as COUP-TFII) and NR2C2 (also known as TR4), which can both bind to these variant repeats [49,50]. NR2F2 or NR2C2 bound to telomeres then recruit ZNF827, a zinc-finger containing protein and subsequently the NuRD protein complex. This presence of the ZNF827-NuRD complex has been reported to remodel the chromatin architecture, creating an environment favorable to recombination and also to help recruit HR proteins to telomeres, protecting them from DDR [51]. Telomeres in other ALT cell lines also bind NR2F2/NR2C2 but the density of the (TCAGGG) binding sites varies considerably between cell lines [52] consequently the role of ZNF827-NuRD complex in telomere lengthening is unclear. NR2F2 and NR2C2 have also been shown to recruit FANCD2 to ALT telomeres, a protein from the Fanconi anemia (FA) repair pathway usually involved in DNA interstrand cross-link repair [53]. At ALT telomeres, FANCD2 recruits the endonuclease MUS81 to induce DDR, followed by the loading of the PCNA-POLD3 replication complex. The two orphan nuclear receptors are also thought to promote the insertion of telomeric DNA in specific genomic regions, a phenomenon termed targeted telomere insertion (TTI), which could contribute to the genomic instability [54]; however, it must be noted once again that this was only observed in the WI38-VA13/2RA cell line. Nonetheless, there is recent evidence of TVRs interacting with another protein, the zinc finger and BTB domain-containing ZBTB10. ZBTB10 can bind the canonical telomeric repeat TTAGGG but it was found to be enriched at TTGGGG variant repeats in U2OS cells [55]. ZBTB10 has been shown to interact with the Shelterin subunits TRF2 and RAP1 but its exact role at ALT telomeres has yet to be defined. We propose that telomere erosion, driven by replication or t-loop excision, could lead to exposure of TVRs at the end of the telomere. This could trigger binding of non-Shelterin component proteins, such as NR2F2, NR2C2, ZBTB10, or other zinc-finger containing proteins, that may then modify the DDR at these short telomeres.

## 4. Pathways to Telomere Elongation in ALT and Relationship to Genome Instability

ALT involves a specialized form of homology dependent recombination (HDR) that most closely resembles break-induced-replication (BIR), first described in yeast [56,57]. ALT involves strand invasion of the telomere to be lengthened (recipient) into another telomere (donor) that will be copied. The donor or template DNA could be the sister-telomere, another telomere, or extrachromosomal telomeric DNA. This was first demonstrated in human ALT cells by introducing a plasmid DNA tag within a telomere, which was then copied to another telomere [58] and by detection of recombinant-variant repeat interspersion patterns in telomeres in ALT positive cell lines and tumors but not in telomerase positive cell lines [47]. This is followed by dissolution of the recombination intermediates and synthesis of the complementary strand [59].

Many of the DNA repair and replication proteins necessary for ALT were first discovered through their association with APBs, such as the MRN complex, which is involved in the detection and repair of DSBs [33], as well as the replication protein A (RPA) and the recombinases RAD51 and RAD52 [34]. Evidence for the role of other proteins has been emerging in the past few years: The DNA polymerase delta subunit 3, which appears to be essential only for BIR but dispensable for gene conversion and replication [60]; the RecQ helicases WRN and BLM [61] with a recently found interaction in ALT cells only between the BLM/TOP3A/RMI1/2 (BTR) complex and the Fanconi anemia complementation group M (FANCM) ATPase/translocase [62,63]; finally, proteins usually active in meiosis, such as the heterodimer HOP2-MND1, [64] were found to be expressed in both telomerase and ALT positive cells, but co-localized to telomeres in ALT cells only.

**Figure 2:**
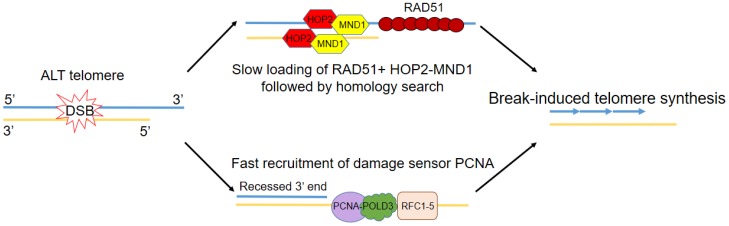
Fast and slow routes to break-induced telomere synthesis. Following double strand break response and end resection at ALT telomeres, two different pathways can be invoked, both leading to break-induced telomere synthesis. One involves the recruitment of RAD51 and HOP2-MND1 to the resected end, followed by RAD51-mediated homology search. This process, however, is believed to be slower than the second pathway, which involves the faster loading of the replication factor C (RFC)-mediated proliferating cell nuclear antigen PCNA, acting as an early sensor of telomere damage. PCNA then recruits Pol δ, with its subunit POLD3 crucial for telomere elongation.

Most recently, the emphasis has been on how different pathways to telomere elongation in ALT involve break-induced telomere synthesis, a mechanism similar to BIR. Rad51-dependent BIR was previously shown to be essential to telomere maintenance in budding yeast strains lacking telomerase [60]. Following DSB response, break-induced telomere synthesis can occur via two different routes: One involves the recruitment of RAD51 and HOP2-MND1 followed by RAD51-mediated homology search [64]. This process, however, is believed to be slower than the second route, which involves the faster loading of the replication factor C (RFC)-mediated proliferating cell nuclear antigen (PCNA), acting as an early sensor of telomere damage [65] (Figure 2). PCNA then recruits Pol δ, with its subunit POLD3 crucial for telomere elongation. RAD52 enables telomeric mitotic DNA synthesis (MiDAs), which is a conservative DNA synthesis mechanism mediated by BIR processes that can lead to genomic duplications [66]. RAD52, however, is dispensable for ALT telomere maintenance, although RAD52-deficient cells were found to be more sensitive to the loss of SLX4, BLM, and FANCD2 [67]. In RAD52-knockout cells, a different BIR pathway involving POLD4, as well as POLD3 is activated, although its full molecular details have yet to be described [68]. Finally, it has been proposed that BIR can be inhibited by the SLX4-SLX1-ERCC4 complex, which resolves the recombination intermediate after telomeric strand invasion and stops telomere extension [69].

Features specific to ALT include the loss of ATRX/DAXX function, a complex involved in chromatin remodeling. ATRX inactivation can be the result of point mutations, small or large deletions but also by mutations not readily detected by sequence analysis such as translocations or promoter silencing mutations, but resulting nonetheless in loss of protein expression [70]. ATRX/DAXX is therefore proposed to be an ALT suppressor and reintroduction of ATRX into cell lines reduced ALT activity [71]. The altered state of telomeric chromatin is indeed a feature of ALT positive cells and could explain the increased expression of telomeric repeat-containing RNAs (TERRA) in ALT positive cells [72] and the increased replication stress found at ALT telomeres. This increased replication stress is responsible for a high level of DDR and telomere dysfunction-induced foci (TIFs), which are sites of DNA damage that can be visualized through their co-localization with γ-H2AX [73]. Interestingly, it has been found that the replication stress response protein SMARCAL1 inhibits ALT activity by allowing for the repair of stalled replication forks at ALT telomeres, thereby preventing a DSB forming from the fork collapse and subsequent BIR promoting telomere synthesis [74].

High levels of genomic instability are seen in immortalized cells that have escaped telomere-driven crisis, a state characterized by end-to-end telomere fusions [4]. Telomere fusions lead to the formation of anaphase bridges, which, once resolved, results in chromothripsis and micronucleus formation [75]. ALT positive cell lines display frequent micronucleation, as well as abnormal karyotypes and copy number alteration [70] and this could suggest that ALT is activated after or as cells escape crisis. Nevertheless, the loss of ATRX can also cause chromosome segregation errors and the subsequent formation of micronuclei [76]. It is also likely that the aforementioned targeted telomere insertion (TTI) contributes greatly to genomic instability in ALT cells, with the possibility that the inserted telomeric DNA creates fragile sites that are difficult to replicate and prone to breakage [54].

In sarcomas, ALT has been linked to chromosomal instability in osteosarcomas and chondrosarcomas [77] and to complex karyotypes in soft tissue sarcomas [78,79], with the observation that sarcomas harboring specific translocations were more likely to display a telomere length similar to that of normal, adjacent tissue and have near-normal karyotypes, as opposed to the heterogeneous telomere length and complex karyotypes seen in ALT positive sarcomas. Cytogenetic studies in ten ALT positive cell lines confirmed the high frequency of fragile sites and elevated levels of chromosomal instability [80], another sign that the ALT mechanism appears unable to stabilize whole karyotypes [78], possibly through the high levels of telomere dysfunction caused by elevated replication stress.

## 5. Therapeutic Outlook and Challenges

Some rarer cancers such as sarcomas, which are frequently found to be ALT positive, also appear to be refractory to chemotherapy: For example, myxofibrosarcomas are relatively chemo-insensitive and over 75% of tumor samples were previously found to be ALT positive [11,81]. Novel treatments are therefore needed to improve the poor prognosis usually associated with them. With ALT utilizing the cell’s machinery for DNA repair and replication, targeting the DNA repair or synthesis proteins used in ALT would more than likely mean high levels of toxicity for healthy cells [4]. The MRN complex had previously been recognized as a potential target [33,82] and the compound mirin identified as an MRN and therefore HDR, inhibitor [83]; however, there is currently no data suggesting the drug has progressed to clinical trials. There is, however, continued interest in trying to identify potential ALT-targeted therapies which could have limited adverse effects on normal cells (Table 1), unlike those seen with the telomerase inhibitor Imetelstat. Imetelstat first entered clinical trials in 2005 and has yet to progress beyond phase II (a stage at which the efficiency and side-effects of a drug are still being evaluated), due to patients commonly developing severe hematological side-effects [84] but also because its efficacy seems to be telomere-length dependent. In a phase II study of Imetelstat for advanced non-small cell lung cancer, administration of the drug had no effect on progression-free survival [85]; however, there was an improvement in overall survival in patients whose tumors had shorter telomeres. Recent efforts to look for molecular targets have identified FANCM as indispensable for the proliferation of ALT cells [62,63] but non-essential in normal cells, thereby making its synthetic inhibition toxic to ALT cells only. Other ALT-specific targets of interest include TSYPL5, a protein which inhibits the ubiquitin hydrolase USP7. TSYPL5 is a component of APBs that protects POT1 from poly-ubiquitination and subsequent proteosomal degradation [86]. However, it is not clear why TSYPL5 depletion and the associated degradation of POT1 was toxic to ALT cells only, while the viability of normal or telomerase positive cells was unchanged. The lysine acetyl transferase PCAF is thought to regulate the transcription of genes involved in APB formation; its inhibition with anacardic acid not only decreased ALT activity but also made cells more sensitive to irradiation [87]. This could prove particularly useful in the treatment of ALT positive sarcomas, which usually includes pre or post-surgical radiotherapy.

Arsenic trioxide is already used in the treatment of acute promyelocytic leukemia and as it exerts its antineoplastic effect by binding to the PML protein [88], it also should be considered for use in the treatment of ALT positive cancers. Trabectedin is a marine alkaloid that covalently binds the minor groove of the DNA double helix, eventually triggering a response from DSB repair pathways [89]. ALT cells have been shown to be more sensitive to Trabectedin than telomerase positive cells [90] but the reason for this selective toxicity is unknown. Nonetheless, it must be said that Trabectedin has already been approved for use in soft tissue sarcomas [91], which, along with osteosarcomas, are known to have a higher prevalence of ALT than other tumors.

The most promising compounds, however, may just be those that prevent telomere elongation, regardless of the tumor’s TMM. The G-quadruplex stabilizing compound, 3,6-bis(4-methyl-2-vinylpyrazinium iodine) carbazole (BMVC4), which was originally tested because of its telomerase inhibitory properties, was found to induce senescence in both telomerase and ALT positive cells through the activation of DDR [92]. Since then, several compounds targeting G-quadruplexes have been developed and two of them have reached clinical trial stage [93]. It will be interesting to see whether the Cisplatin-derived agent Tetra-Pt(bpy), which can convert telomeric single-stranded DNA to G-quadruplexes, thereby inhibiting ALT and impairing cell proliferation [94], might have the same effect on telomerase positive cells.

## 6. Concluding Remarks

Rapid developments in the past few years have not only shed light on the molecular pathways involved in ALT, but also helped to identify potential targets for therapies. There are, as always, many questions that remain to be answered, such as, at what stage of tumorigenesis ALT gets activated and why it is more prevalent in some cancers than in others. Moreover, if successful ALT-targeted therapies are to make it to the clinic, one must bear in mind that drug resistance, in the form of telomerase reactivation, could arise, making the search for compounds that target telomere extension, regardless of the TMM, crucial.

## Figures and Tables

**Table 1:** Potential ALT-targeted drugs.

Drug Name	Reference	Mechanism of Action	Comments
Anacardic acid	[87]	Inhibits PCAF, a lysine acetyl transferase involved in regulation of APB-related genes	Natural product, not yet entered clinical trials
Arsenic Trioxide	[88]	Binds PML protein	Approved for use in the treatment of acute promyelocytic leukemia
CX-5461	[93]	G-quadruplex stabilizer	Currently in clinical trial phase 1
Mirin	[33,82,83]	Inhibits MRN	Small molecule inhibitor, has yet to progress to clinical trial stage
PIP-199	[62]	Inhibitor of the MM2-RMI interaction within FANCM-BTR	Small molecule inhibitor, for research use only. Requires further studies before consideration for clinical trials
Quarfloxin	[93]	G-quadruplex stabilizer	Clinical trial terminated in phase 2
Tetra-Pt(bpy)	[94]	Converts telomeric single-stranded DNA to G-quadruplex	Cisplatin derivative, not progressed to clinical trials yet
Trabectedin	[90]	Binds minor groove of DNA double helix	ALT positive cells found to be more sensitive than telomerase positive; reason unknown
